# Time Series Modelling of Syphilis Incidence in China from 2005 to 2012

**DOI:** 10.1371/journal.pone.0149401

**Published:** 2016-02-22

**Authors:** Xingyu Zhang, Tao Zhang, Jiao Pei, Yuanyuan Liu, Xiaosong Li, Pau Medrano-Gracia

**Affiliations:** 1 Department of Epidemiology and Health Statistics, West China School of Public Health, Sichuan University, Chengdu, Sichuan, P.R.China; 2 Department of Anatomy with Radiology, University of Auckland, Auckland, New Zealand; 3 Department of Science and Education, Sichuan Cancer Hospital & Institute, Sichuan, China; Columbia University, UNITED STATES

## Abstract

**Background:**

The infection rate of syphilis in China has increased dramatically in recent decades, becoming a serious public health concern. Early prediction of syphilis is therefore of great importance for heath planning and management.

**Methods:**

In this paper, we analyzed surveillance time series data for primary, secondary, tertiary, congenital and latent syphilis in mainland China from 2005 to 2012. Seasonality and long-term trend were explored with decomposition methods. Autoregressive integrated moving average (ARIMA) was used to fit a univariate time series model of syphilis incidence. A separate multi-variable time series for each syphilis type was also tested using an autoregressive integrated moving average model with exogenous variables (ARIMAX).

**Results:**

The syphilis incidence rates have increased three-fold from 2005 to 2012. All syphilis time series showed strong seasonality and increasing long-term trend. Both ARIMA and ARIMAX models fitted and estimated syphilis incidence well. All univariate time series showed highest goodness-of-fit results with the ARIMA(0,0,1)×(0,1,1) model.

**Conclusion:**

Time series analysis was an effective tool for modelling the historical and future incidence of syphilis in China. The ARIMAX model showed superior performance than the ARIMA model for the modelling of syphilis incidence. Time series correlations existed between the models for primary, secondary, tertiary, congenital and latent syphilis.

## Introduction

Syphilis is a sexually transmitted infection caused by the spirochete bacterium *Treponema pallidum* subspecies *pallidum*. Syphilis can present in one of four different stages: primary, secondary, latent, and tertiary, and may also occur congenitally [1, 2]. The rate of syphilis has increased dramatically in recent decades, becoming both a burden and a threat to public health. The reported total syphilis rate in China was 0.2/100,000 in 1993, whereas primary and secondary syphilis represented 5.7/100,000 in 2005 [3]. In 2008, an average of more than 1 baby per hour was born with congenital syphilis in China, with a total of 9480 cases [4]. Syphilis is among the top five reported communicable diseases in many major municipalities and provinces of China [5]. It can cause serious health problems, increasing the risk of HIV transmission by two to five times [6]. The increasing incidence of syphilis represents a growing concern to medical providers, policy makers and community populations. To help devise a health plan to decrease syphilis’ occurrence and associated patient burden, it is therefore important to adequately strengthen its surveillance and study its incidence and behavior.

Time series methods have been widely used to analyze infectious diseases’ surveillance data in recent decades, including data for sexually transmitted diseases. Different time series models were used to forecast the epidemic behavior in previous studies [7, 8]. For example, decomposition methods were used to forecast nine notifiable infectious diseases in China [7]. Autoregressive integrated moving average models (ARIMA) are widely applied in infection time series modelling including tuberculosis [9] typhoid fever [10], gonorrhea [11] and hepatitis [12]. Autoregressive conditional heteroskedasticity and generalized autoregressive conditional heteroskedasticity models have been used to investigate the risk factors associated with syphilis [13]. Multi-variable time series analysis were used to explore the association between malaria’s behavior and weather patterns [14].

Decomposition methods are typically used to analyze the seasonal and long-trend patterns of a disease [7]: the seasonal behavior is extracted as a seasonal index whereas the secular trend is expressed with a linear regression model. ARIMA models [15] [16] are widely used to model the time series based on a differencing process and an autoregressive and moving average (ARMA) model. The ARMA model views the value at time *t* as a linear combination of its previous values and residuals. The ARIMAX model is a multi-variable time series analysis method, which includes an additional covariate in the ARIMA model. The model tries to forecast the infection time series using an exogenous covariate series. The method is widely used to explore the influence of covariate factors in the infection behavior. For example, the model has been used to explore the association of the malaria behavior with temperature, rainfall and humidity [14]. The model was also applied to explore the association of the human and bovine brucellosis series [17]. However, there are fewer studies focused on time series modelling of different sub-types of syphilis, as well as their time series correlation [15].

The aim of the study is to explore the univariate and multivariate time series characteristics of primary, secondary, tertiary, latent and congenital syphilis. Incidence data of general syphilis, primary syphilis, secondary syphilis, tertiary syphilis, latent syphilis and congenital syphilis of China from 2005 to 2012 were used. A statistical decomposition method was used to explore the seasonality and the long trend of the behavior of the different types of syphilis. The ARIMA was modelled on the general syphilis univariate time series. The ARIMAX model was fitted to explore the time series association among primary, secondary, latent, tertiary and congenital syphilis.

## Data and Methods

**Fig 1:**
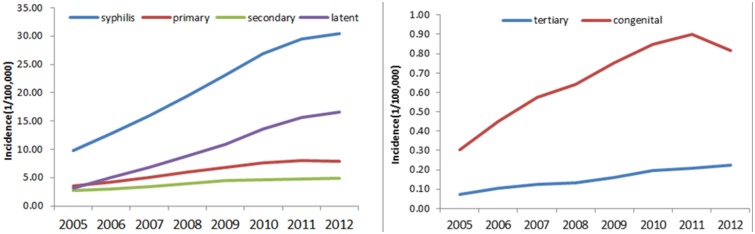
Trend of syphilis incidence in China from 2005–2012 by year

In this study, we used publicly available data from the Public Health Scientific Data website (http://www.phsciencedata.cn/Share/en/index.jsp). The data are collected and reported by the Chinese Centre for Disease Prevention and Control (CDC). Specifically we used the monthly incidence of syphilis. The incidence data of syphilis, primary syphilis, secondary syphilis, tertiary syphilis, latent syphilis and congenital syphilis from 2005 to 2012 were collected by the Chinese National Surveillance System firstly established in 2004 and are available through the CDC. Since the new reporting system was established, more cases have been able to be identified and data have become more complete and reliable [18]. A survey showed that the average omission rate (percentage of unreported cases) was 13% among the medical institutions throughout the country. The compliance rate (percentage of the compliant recordings) of out-patient daily registration was 96%, the registry integrity rate (percentage of reported cases with complete information) was 97%, and the timely report rate (percentage of reported cases within CDC’s time limits) of medical institutions was 91% [19]. There are 39 notifiable infectious diseases included in the surveillance system, which are divided into Classes A, B, and C [20]. Class A notifiable diseases include the plague and cholera which can cause large epidemics in a very short time. Class B notifiable diseases include 26 infectious diseases that might cause epidemics, such as AIDS, viral hepatitis, polio, rabies, dengue fever, anthrax, scarlet fever, brucellosis, gonorrhea, syphilis, etc. Class C notifiable diseases include 11 less severe and less infectious diseases such as mumps, rubella, acute hemorrhagic conjunctivitis, leprosy, leishmaniasis, hydatid disease, etc. Syphilis is thus currently one of the class B notifiable diseases in China. According to Chinese law, once syphilis patients or suspected patients are detected, it must be reported to the system within 12 hours in urban areas and 24 hours in rural areas. The trend of syphilis incidence in China from 2005–2012 is shown in Fig 1. Rates of syphilis incidence have increased from 2005 to 2012. The rate of reported syphilis in China was 9.73 cases per 100,000 people in 2005, 12.80 in 2006, 15.88 in 2007, 19.49 in 2008, 23.07 in 2009, 26.86 in 2010, 29.47 in 2011 and 30.44 in 2012. Latent syphilis is the most common type, accounting for 54.64% of total number of syphilis cases in 2012. Primary syphilis is the second, accounting for 26.02%, secondary syphilis 15.92%, congenital syphilis 2.68% and tertiary 0.74%. Syphilis incidence increases dramatically from 2005 to 2012. The time series of syphilis shows a seasonal trend, with higher incidence in summer and lower incidence in winter.

### Decomposition methods

Generally, the decomposition method attempts to decompose the two underlying patterns, long term trend and seasonality, characterizing the infectious behavior in the time series data:

Time series = Seasonality + Long term trend + Residual

The seasonality of syphilis’ incidence can be expressed by calculating the seasonal indices. To calculate these, overall incidences are averaged first, and then the averaged incidence is divided by the mean incidence for each month [7]. The seasonal index is between 0 and 1, when the incidence is below the average level. Otherwise, the value will be greater than 1. After deriving the seasonal indices, the seasonality of the series should be removed by dividing by the corresponding index. A linear regression model is fitted to present the long-term trend of the incidence. The linear relationship between the deseasonalized syphilis’ incidence (dependent variable) and time *t* (independent variable) is estimated in the model.

### ARIMA Model

The ARIMA model is one of the most popular univariate time series models, which is widely used in infectious disease modelling. As is described in previous studies [7, 10], the ARIMA model is established based on the ARMA models, expressing the current syphilis incidence linearly with its previous incidence (autoregressive) and the residual series (moving average). The ARMA model can be expressed as:

$$y(t)=\phi_{1}y(t-1)+\ldots +\phi_{p}y(t-p)+\varepsilon (t)-\theta_{1}\varepsilon (t-1)-\ldots -\theta_{q}\varepsilon (t-q)$$

where *y*(*t*) refers to the value of the time series at time *t* and *ε*(*t*) is the residual at time *t*. *ϕ* and *θ* are their corresponding coefficients. The ARIMA model deals with non-stationary time series using a differencing process based on the ARMA model. The model can be expressed as ARIMA (*p*, *d*, *q*) × (*P*, *D*, *Q*)_*s*_, where *p*, *d*, and *q* are non-negative integers that refer to the order of the autoregressive, integrated, and moving average parts of the model respectively whereas *P*, *D* and *Q* represent the order of the seasonal autoregressive, differencing and moving average respectively (not shown in the above equation). The subscripted letter “*s*” shows the seasonal period length.

The ARIMA modeling procedure introduced by Box and Jenkins, consists of three iterative steps: identification, estimation, and diagnostic checking [21]. Before the identification step, data must be stationary, which can be achieved by performing an appropriate seasonal difference in addition to the regular difference of the ARIMA model. Stationarity can be tested using the Augmented Dickey-Fuller (ADF) method [22]. The identification step includes the process of determining seasonal and non-seasonal orders using the autocorrelation functions (ACF) and partial autocorrelation functions (PACF) of the differenced series. Parameters in the ARIMA model(s) are estimated using the conditional least square (CLS) method after the identification step [23]. Finally, the adequacy of the established model for the series is verified by employing white noise tests [24] to check whether the residuals are independent and normally distributed. It is possible that several ARIMA models may be identified, and the selection of an optimum model becomes necessary. The optimum model is usually determined based on the Akaike Information Criterion (AIC) and Schwartz Bayesian Criterion (SBC) [25]. The AICc is also calculated based on AIC, which is a correction for finite sample sizes [26].

### ARIMAX

In contrast with ARIMA where the previous values of the dependent variable (AR) and residual series (MA) are taken after the differencing process, the ARIMAX model is an extension of ARIMA modelling incorporating an explanatory independent variable. An ARMAX model can simply be viewed as a multiple regression with one or more AR and MA terms. The ARIMAX model can be expressed as:

$$y(t)=\beta x(t)+\phi_{1}y(t-1)+\ldots +\phi_{p}y(t-p)+\varepsilon (t)-\theta_{1}\varepsilon (t-1)-\ldots -\theta_{q}\varepsilon (t-q)$$

where *x*(*t*) is a covariate at time *t* and *β* is its associated coefficient. *Y*(*t* − 1)…*Y*(*t* − *p*) are the previous values, and *ε*(*t*)…*ε*(*t* − *q*)are the residual series. For each type of syphilis incidence, all the other types of syphilis incidence were used as the covariate variables. The covariates which were not significant (p>0.05) in the model were removed afterwards. As in ARIMA models, several models may be identified in the ARIMAX framework. The selection of the optimum ARIMAX model is also based on AIC (AICc) and SBC.

Before fitting the ARIMAX model, cross-correlations between different syphilis time series are calculated [27]. The ARIMAX model hypothesizes that the time series *y*(*t*) is related to past lags of the *x*(*t* + *m*) series. The cross correlation function (CCF) is defined as the linear correlation between *x*(*t* + *m*) and *y*(*t*), for m = 0, ±1, ±2, ±3, etc. CCF can help to identify lags of the x-variable that might be useful predictors of *y*(*t*) [28]. The value of CCF is between −1 and +1. Absolute CCF values closer to 1 mean higher correlation between the two series.

ARIMAX models were fitted to the primary, secondary, tertiary, latent and congenital syphilis series from the year 2005 to 2011 and tested by estimating the syphilis incidence for 2012. The covariate variables as well as their interaction terms were determined first by including all the covariates and the nitration term in the model and then excluding those which were not statistically significant. ARIMA Procedure in *SAS 9*.*3* was used to fit the ARIMA and ARIMAX model. The estimation for incidence in 2012 was generated using rolling prediction step by step.

## Results

### Seasonal indices

**Fig 2:**
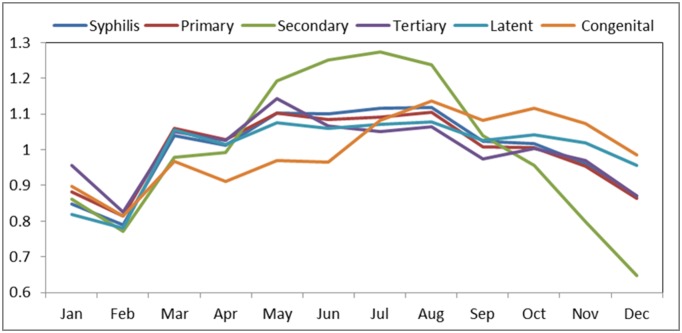
Seasonal indices of each type of syphilis

Seasonal indices of primary, secondary, tertiary, latent and congenital syphilis were obtained from the original incidence series (Table 1 and Fig 2). The seasonal indices show the seasonality of the incidence behavior of each syphilis series. Generally, syphilis shows higher incidence in summer (June, July, August) than in winter (December, January, February). Primary, secondary, tertiary and latent syphilis peak in May to August. However, the peak of the congenital case is deferred when compared to others, from July to November. The extent of the seasonality differs by type. The seasonality of the secondary syphilis is more pronounced. The long-trend of each disease can be shown from the linear regression model for the original syphilis incidence removed of seasonality. The estimation of the coefficient, constant, *R*^*2*^ and *p* values for the regression model is shown in Table 2. *R*^*2*^ is the coefficient of determination ranging from 0 to 1. An *R*^*2*^ closer to 1 means that the regression model fits the data with minimal error, while an *R*^*2*^ near 0 means the regression model does not fit the data well. From the coefficient of the regression, we can derive how much the incidence changes on average by month; for instance, for latent syphilis the incidence changing rate increases on average by 0.01390/100,000 every month after having removed the effect of seasonality.

**Table 1:** Syphilis incidence seasonal indices.

	Jan	Feb	Mar	Apr	May	Jun	Jul	Aug	Sep	Oct	Nov	Dec
Primary syphilis	0.88	0.81	1.06	1.03	1.10	1.08	1.09	1.10	1.01	1.00	0.95	0.86
Secondary syphilis	0.86	0.77	0.98	0.99	1.19	1.25	1.27	1.24	1.04	0.96	0.80	0.65
Tertiary syphilis	0.96	0.82	1.05	1.02	1.14	1.07	1.05	1.06	0.97	1.00	0.97	0.87
Latent syphilis	0.82	0.78	1.06	1.01	1.08	1.06	1.07	1.08	1.03	1.04	1.02	0.96
Congenital syphilis	0.90	0.81	0.97	0.91	0.97	0.96	1.08	1.14	1.08	1.12	1.07	0.98

**Table 2:** Linear regression model for each syphilis time series removed seasonality.

	Constant	Coefficient	Coefficient 95% confidence Interval		R^2^	P
Primary syphilis	0.28232	0.00473	0.00442	0.00504	0.908	0.002
Secondary syphilis	0.21493	0.00237	0.00219	0.00255	0.872	<0.001
Tertiary syphilis	0.00550	0.00015	0.00014	0.00016	0.903	<0.001
Latent syphilis	0.16333	0.01390	0.01329	0.01451	0.955	0.007
Congenital syphilis	0.02824	0.00055	0.00050	0.00060	0.848	<0.001

Note: The linear relationship between the deseasonalized syphilis incidence (dependent variable) and time *t* (independent variable) is estimated in the model.

### ARIMA models

ARIMA models were established to the 5 syphilis time series from the year 2005 to 2011. The model was tested by estimating the values for 2012. The order of the autoregressive and moving average are determined based on the autocorrelation function (ACF) and partial autocorrelation function (PACF) [10]. Several ARIMA models were fitted and the results of the estimations for the 5 syphilis incidence time series are shown in Table 3. The final ARIMA model selected was highlighted according to the criterion of minimum AIC (AICc) and SBC. The 5 syphilis series are in the same ARIMA pattern, and the ARIMA(0,0,1)×(0,1,1) model is consistently the best model for each series. It means that after seasonal differencing, the incidence at time *t* is highly relevant to the residual at time *t*, *t-1*, *t-12* and *t-13*.

**Table 3:** Available ARIMA models fitted for each syphilis series.

Syphilis	Model	AIC	AICC	SBC
Primary Syphilis	ARIMA(0,0,0)×(0,1,0)	-220.21	-220.21	-220.21
	ARIMA(1,0,0)×(0,1,0)	-240.1	-240.04	-237.83
	ARIMA(0,0,1)×(0,1,0)	-264.49	-264.43	-262.23
	ARIMA(1,0,1)×(0,1,0)	-240.1	-240.04	-237.83
	ARIMA(1,0,0)×(0,1,1)	-262.03	-261.86	-257.5
	ARIMA(0,0,1)×(1,1,0)	-273.28	-273.11	-268.76
	ARIMA(1,0,0)×(1,1,0)	-251.45	-251.28	-246.93
	**ARIMA(0,0,1)×(0,1,1)**	**-278.38**	**-278.21**	**-273.85**
	ARIMA(2,0,0)×(0,1,0)	-248.59	-248.24	-244.06
	ARIMA(3,0,0)×(0,1,0)	-257.4	-257.05	-250.61
Secondary Syphilis	ARIMA(0,0,0)×(0,1,0)	-299.15	-299.15	-299.15
	ARIMA(1,0,0)×(0,1,0)	-317.33	-317.27	-315.07
	ARIMA(0,0,1)×(0,1,0)	-335.32	-335.26	-333.05
	ARIMA(1,0,0)×(1,1,0)	-337.25	-337.08	-332.72
	**ARIMA(0,0,1)×(0,1,1)**	**-350.74**	**-350.57**	**-346.22**
	ARIMA(1,0,0)×(0,1,1)	-340.42	-340.25	-335.9
	ARIMA(0,0,1)×(1,1,0)	-350.34	-350.17	-345.81
	ARIMA(2,0,0)×(0,1,0)	-323.53	-323.36	-319
	ARIMA(3,0,0)×(0,1,0)	-331.74	-331.39	-324.95
Tertiary Syphilis	ARIMA(0,0,0)×(0,1,0)	-656.31	-656.31	-656.31
	ARIMA(1,0,0)×(0,1,0)	-680.65	-680.59	-678.38
	ARIMA(0,0,1)×(0,1,0)	-686.42	-686.36	-684.16
	ARIMA(1,0,0)×(1,1,0)	-689.92	-689.75	-685.4
	**ARIMA(0,0,1)×(0,1,1)**	**-709.78**	**-709.61**	**-705.26**
	ARIMA(1,0,0)×(0,1,1)	-702.65	-702.48	-698.13
	ARIMA(1,0,0)×(0,1,1)	-649.36	-649.19	-689.84
	ARIMA(2,0,0)×(0,1,0)	-682.91	-682.74	-678.38
Latent Syphilis	ARIMA(0,0,0)×(0,1,0)	-129.69	-129.69	-129.69
	ARIMA(1,0,0)×(0,1,0)	-162.44	-162.38	-160.18
	ARIMA(0,0,1)×(0,1,0)	-177.83	-177.77	-175.57
	ARIMA(1,0,0)×(1,1,0)	-182.03	-181.86	-177.5
	**ARIMA(0,0,1)×(0,1,1)**	**-193.84**	**-193.67**	**-189.31**
	ARIMA(1,0,0)×(0,1,1)	-184.44	-184.27	-179.92
	ARIMA(1,0,0)×(0,1,1)	-193.77	-193.6	-189.25
	ARIMA(2,0,0)×(0,1,0)	-166.47	-166.3	-161.95
	ARIMA(3,0,0)×(0,1,0)	-177.47	-177.12	-170.68
Congenital Syphilis	ARIMA(0,0,0)×(0,1,0)	-519.28	-519.28	-519.28
	ARIMA(1,0,0)×(0,1,0)	-537.62	-537.56	-535.36
	ARIMA(0,0,1)×(0,1,0)	-558.88	-558.82	-556.62
	ARIMA(1,0,0)×(1,1,0)	-546.76	-546.59	-542.23
	**ARIMA(0,0,1)×(0,1,1)**	**-575.4**	**-575.23**	**-570.88**
	ARIMA(1,0,0)×(0,1,1)	-559.86	-559.69	-555.33
	ARIMA(1,0,0)×(0,1,1)	-565.61	-565.44	-561.09
	ARIMA(2,0,0)×(0,1,0)	-545.65	-545.3	-541.13
	ARIMA(3,0,0)×(0,1,0)	-552.22	-551.87	-545.43

Note: the first model for each syphilis ARIMA(0,0,0)×(0,1,0) only included the seasonal differencing term. The second model ARIMA(1,0,0)×(0,1,0), i.e., SAR(1) included the seasonal differencing and an autoregressive term. The third model ARIMA(0,0,1)×(0,1,0), i.e., SMA(1), included the differencing and moving average terms. These three models can be treated as the baseline. The final ARIMA model selected is highlighted in bold.

### Cross-correlations

**Fig 3:**
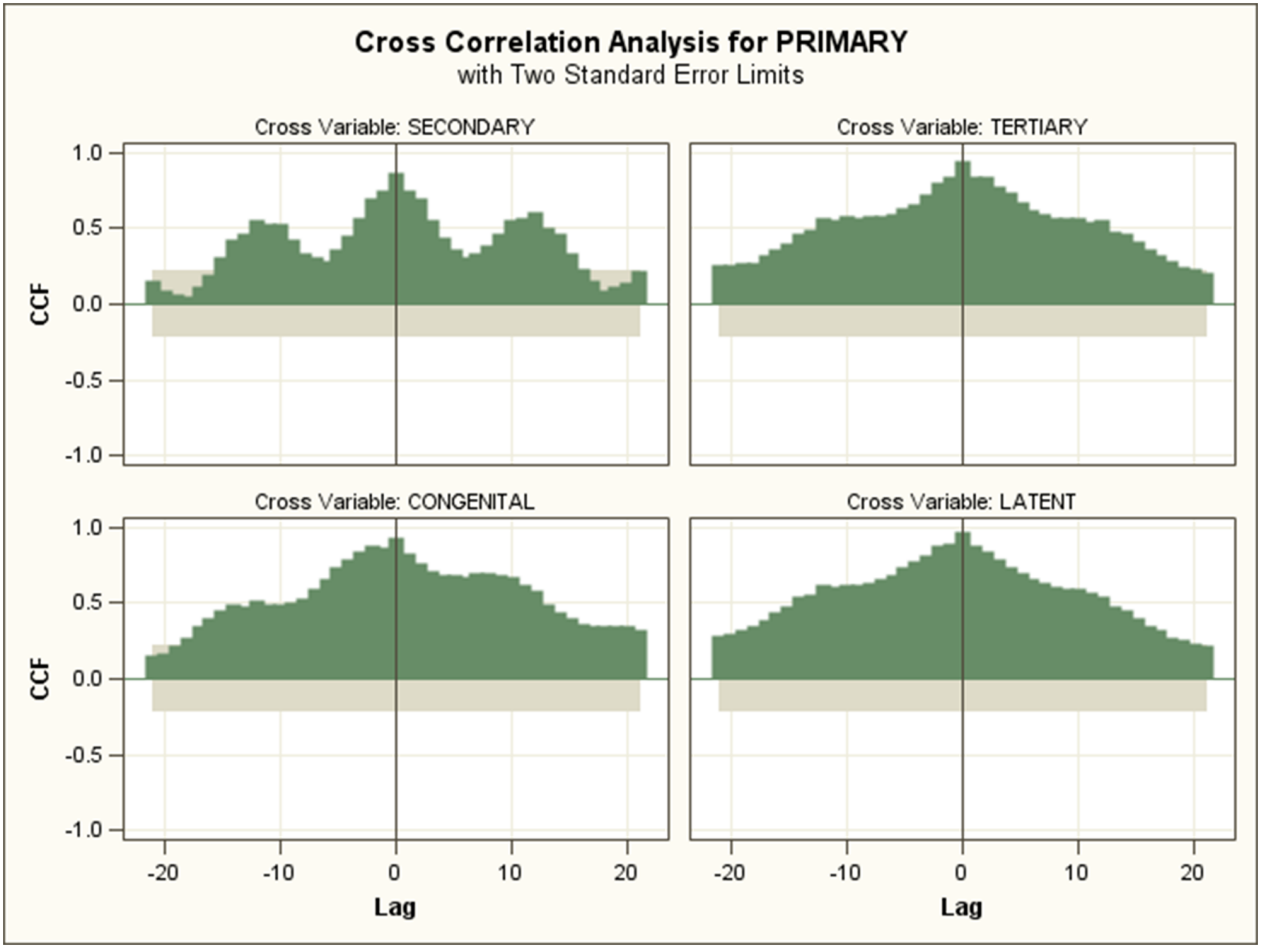
Cross correlation analysis between primary syphilis and other syphilis time series (highest values appear when the lag is 0)

**Fig 4:**
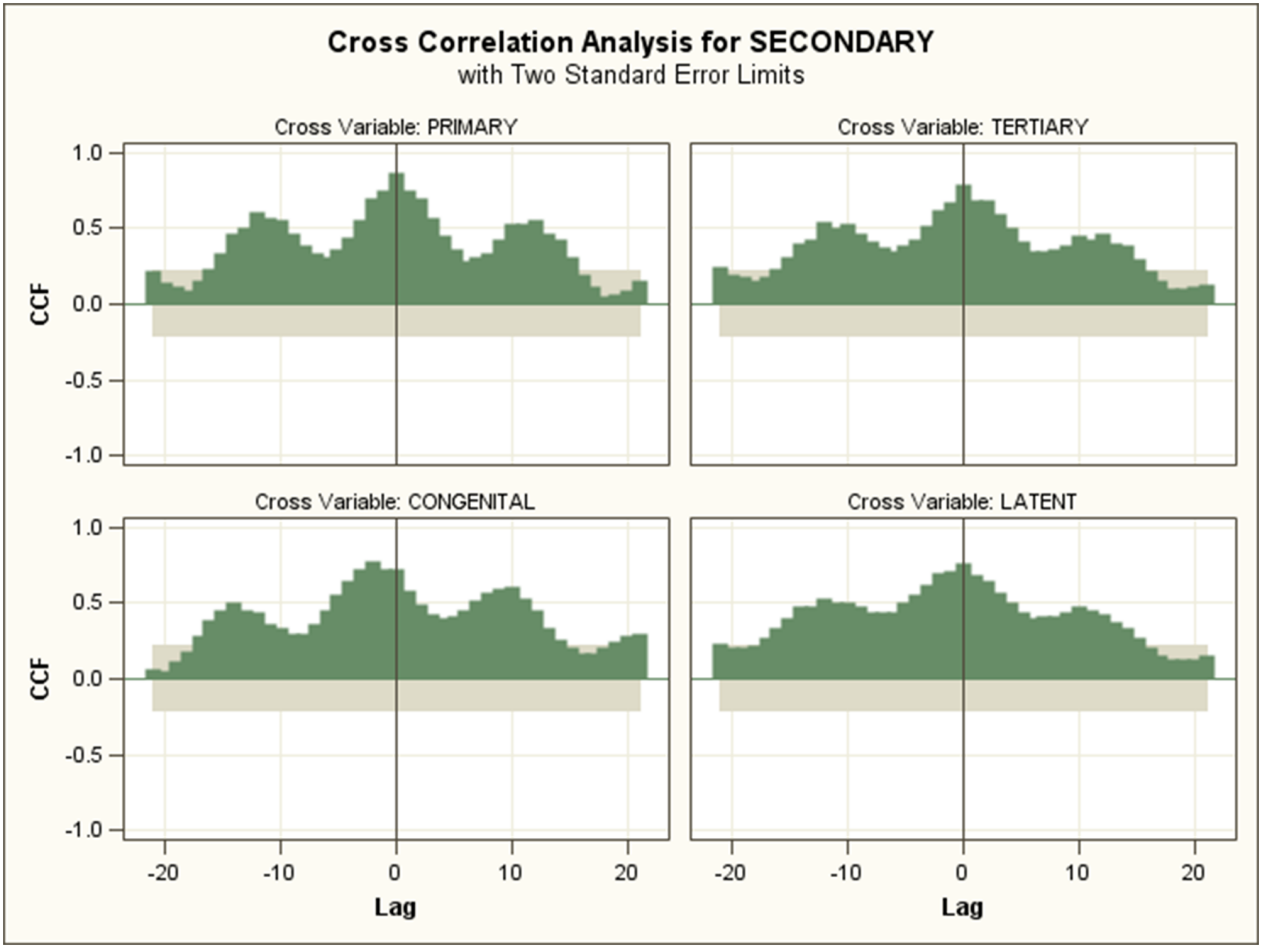
Cross correlation analysis between secondary syphilis and other syphilis time series (highest values appear when the lag is 0 except between secondary and congenital syphilis).

Cross-correlations between different syphilis were calculated and the highest CCF values and the lags are shown in Table 4. The cross-correlation between primary, secondary and other series are shown as an example in Figs 3 and 4. Highest values appear when the lags are 0 except between secondary and congenital syphilis. In general, CCF values were high, with primary, tertiary, congenital and latent syphilis over 0.90, while CCF values of secondary syphilis with others were slightly slower.

**Table 4:** The highest cross-correlation between syphilis incidence time series (the values in the brackets are the lags, the lags are 0 except between secondary and congenital syphilis).

	Primary	Secondary	Tertiary	Congenital	Latent
Primary	1	0.87(0)	0.95(0)	0.93(0)	0.97(0)
Secondary		1	0.79(0)	0.77(-2)	0.76 (0)
Tertiary			1	0.90(0)	0.95(0)
Congenital				1	0.96(0)
Latent					1

### ARIMAX models

**Fig 5:**
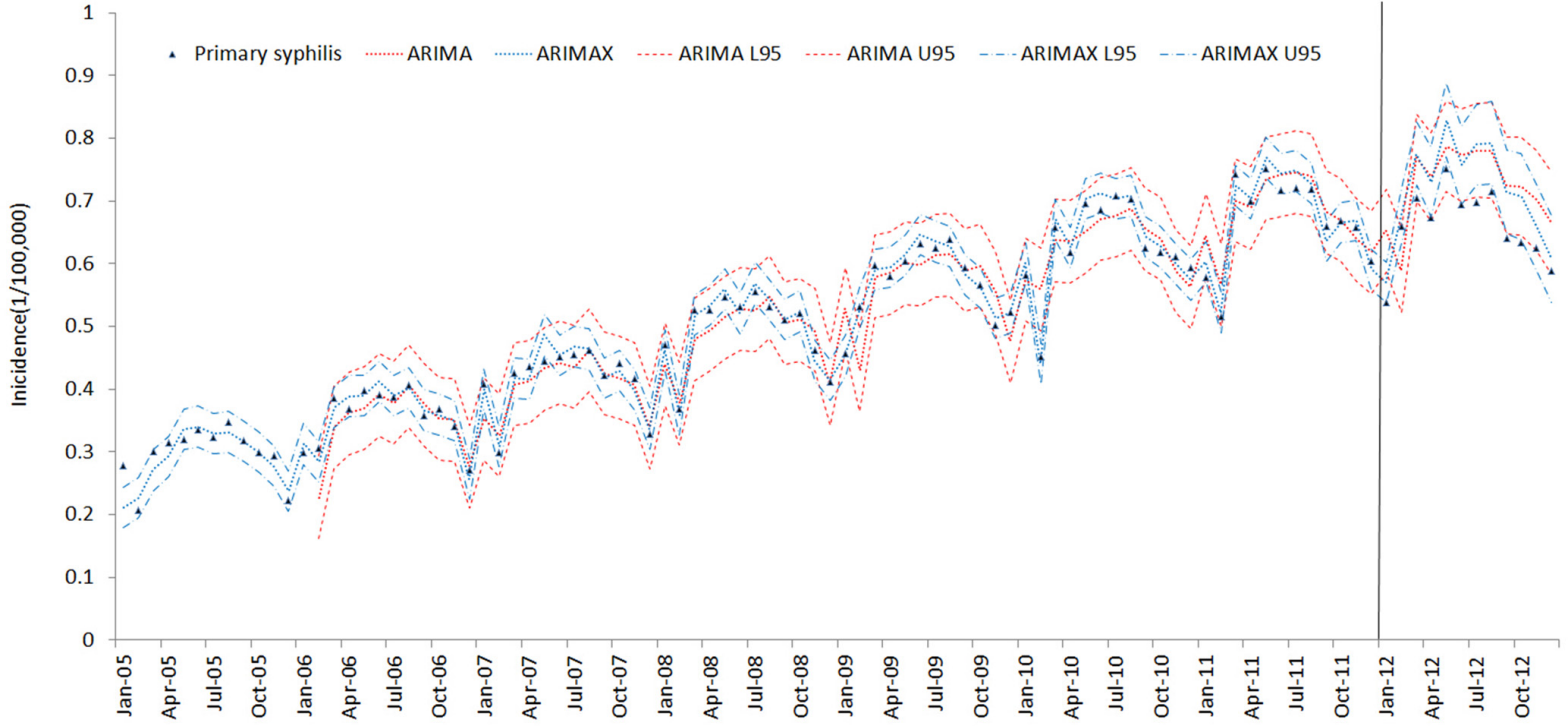
Primary syphilis incidence fitting and testing performance by ARIMA and ARIMAX (U95 and L95 refer to the upper and lower 95% confidential interval respectively. The vertical gray line separates modelling from estimates.)

**Fig 6:**
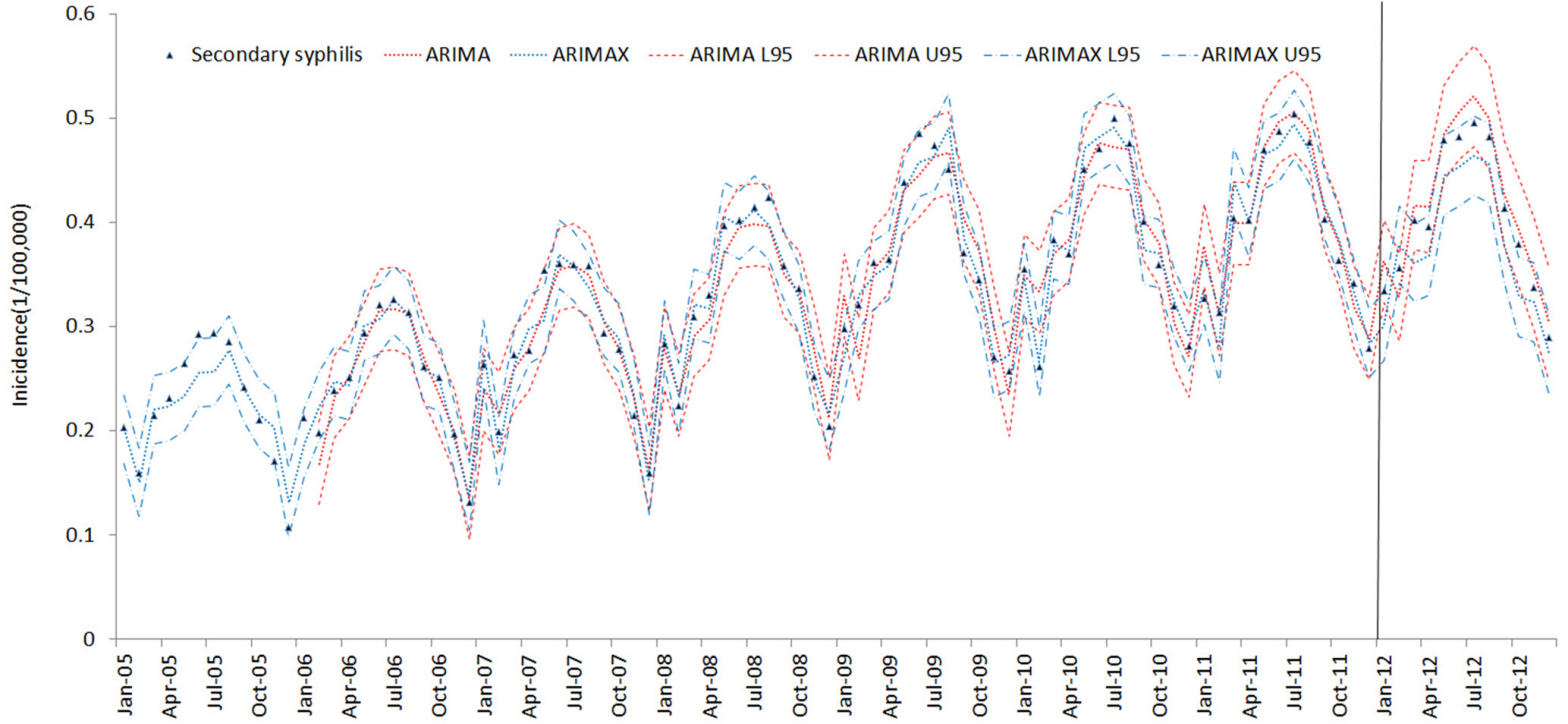
Secondary syphilis incidence fitting and testing performance by ARIMA and ARIMAX (U95 and L95refers to the upper and lower 95% confidential interval respectively. The data were divided into modeling and forecasting groups with a vertical line; the left is the modeling part, and the right is the forecasting part.)

**Fig 7:**
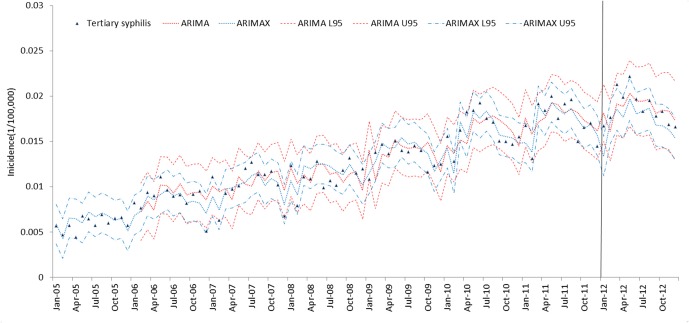
Tertiary syphilis incidence fitting and testing performance by ARIMA and ARIMAX (U95 and L95refers to the upper and lower 95% confidential interval respectively. The data were divided into modeling and forecasting groups with a vertical line; the left is the modeling part, and the right is the forecasting part.)

**Fig 8:**
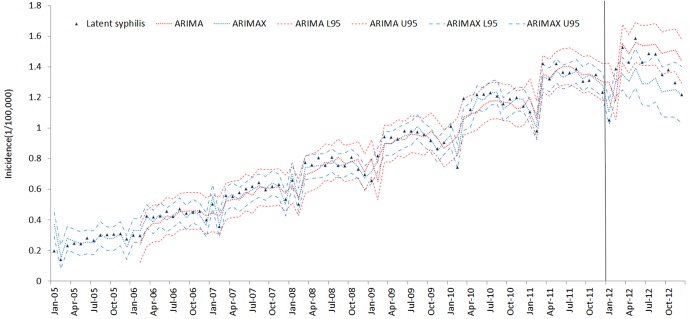
Latent syphilis incidence fitting and testing performance by ARIMA and ARIMAX (U95 and L95refers to the upper and lower 95% confidential interval respectively. The data were divided into modeling and forecasting groups with a vertical line; the left is the modeling part, and the right is the forecasting part.)

**Fig 9:**
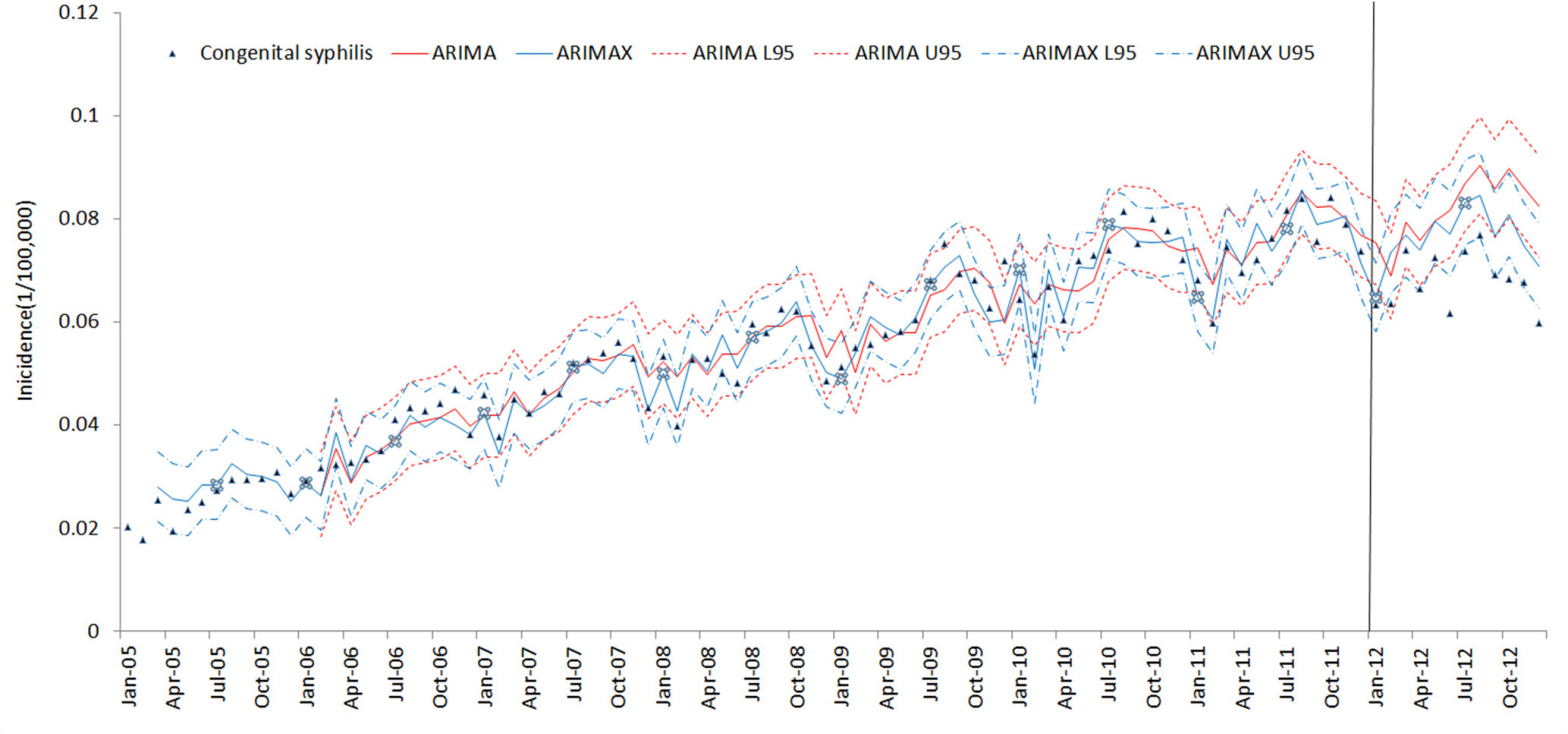
Congenital syphilis incidence fitting and testing performance by ARIMA and ARIMAX (U95 and L95refers to the upper and lower 95% confidential interval respectively. The data were divided into modeling and forecasting groups with a vertical line; the left is the modeling part, and the right is the forecasting part.)

The results of the ARIMAX models for the 5 types of syphilis incidence time series are presented in Table 5. The best ARIMA model is highlighted in Table 5 according to the AIC (AICc) and SBC criteria. ARIMAX obtained smaller AIC (AICc) and SBC than the corresponding univariate ARIMA models, which indicates that the fitting performance improved with the inclusion of the covariate variables. The fitting and prediction of incidence for the ARIMA and ARIMAX models over six years are plotted in Figs 5–9. Syphilis’ incidence appears well fitted by the two models.

**Table 5:** Estimation of available ARIMAX models for each series.

Syphilis	Model	Covariates	AIC	AICC	SBC
Primary	ARIMAX(0,0,0)×(0,0,0)	Secondary, Tertiary, Latent, Secondary*Tertiary	-451.83	-451.32	-442.1
	ARIMAX(1,0,0)×(0,0,0)	Secondary, Tertiary, Latent, Secondary*Tertiary	-469.62	-468.85	-457.47
	ARIMAX(0,0,1)×(0,0,0)	Secondary, Tertiary, Latent, Secondary*Tertiary	-461.87	-461.1	-449.72
	**ARIMAX(0,0,1)×(1,0,0)**	Secondary, Tertiary, Latent, Secondary*Tertiary	**-472.02**	**-470.93**	**-457.43**
	ARIMAX(1,0,0)×(0,0,1)	Secondary, Tertiary, Latent, Secondary*Tertiary	-470.54	-469.45	-455.95
	ARIMAX(0,0,0)×(1,0,1)	Secondary, Tertiary, Latent, Secondary*Tertiary	-470.54	-469.45	-455.95
	ARIMAX(1,0,0)×(1,0,1)	Secondary, Tertiary, Latent, Secondary*Tertiary	-471.67	-470.2	-454.65
Secondary	ARIMAX(0,0,0)×(0,0,0)	Primary, Latent, Primary*Latent	-346.31	-346.01	-339.02
	ARIMAX(1,0,0)×(0,0,0)	Primary, Latent, Primary*Latent	-376.06	-375.55	-366.34
	ARIMAX(0,0,1)×(0,0,0)	Primary, Latent, Primary*Latent	-376.23	-375.73	-366.51
	**ARIMAX(1,0,0)×(1,0,0)**	Primary, Latent, Primary*Latent	**-447.15**	**-446.38**	**-435**
	ARIMAX(1,0,1)×(0,0,0)	Primary, Latent, Primary*Latent	-381.75	-380.98	-396.6
	ARIMAX(,0,1)×(0,0,1)	Primary, Latent, Primary*Latent	-401.33	-400.56	-389.17
	ARIMAX(1,0,0)×(0,0,1)	Primary, Latent, Primary*Latent	-402.89	-402.12	-390.74
	ARIMAX(0,0,1)×(1,0,0)	Primary, Latent, Primary*Latent	-441.6	-440.83	-429.44
Tertiary	ARIMAX(0,0,0)×(0,0,0)	Primary, Latent	-892.12	-891.97	-887.26
	**ARIMAX(1,0,0)×(0,0,0)**	**Primary, Latent**	**-900.95**	**-900.65**	**-893.66**
	ARIMAX(1,0,(3))×(0,0,0)	Primary, Latent	-902.55	-902.04	-892.83
	ARIMAX(0,0,(3))×(0,0,0)	Primary, Latent	-897.93	-897.63	-890.64
	ARIMAX(0,0,1)×(0,0,0)	Primary, Latent	-897.57	-897.27	-890.27
Latent	ARIMAX(0,0,0)×(0,0,0)	Primary, Secondary, Tertiary, Congenital, Primary*Secondary, Secondary*Congenital, Tertiary*Congential	-284.93	-283.46	-267.91
	ARIMAX(0,0,1)×(0,0,0)	Primary, Secondary, Tertiary, Congenital, Primary*Secondary, Secondary*Congenital, Tertiary*Congential	-283.52	-281.6	-264.08
	ARIMAX(0,0,0)×(1,0,0)	Primary, Secondary, Tertiary, Congenital, Primary*Secondary, Secondary*Congenital, Tertiary*Congential	-253.77	-251.85	-234.32
	**ARIMAX(0,0,0)×(1,0,0)**	Primary, Secondary, Tertiary, Congenital, Primary*Secondary, Secondary*Congenital, Tertiary*Congential	**-348.89**	**-346.97**	**-327.01**
	ARIMAX(0,0,0)×(0,0,1)	Primary, Secondary, Tertiary, Congenital, Primary*Secondary, Secondary*Congenital, Tertiary*Congential	-304.83	-302.91	-285.39
Congenital	ARIMAX(0,0,0)×(0,0,0)	Primary,Secondary, Latent, Secondary*Latent	-672.88	-672.37	-663.25
	ARIMAX(0,0,1)×(0,0,0)	Primary,Secondary, Latent, Secondary*Latent	-682.67	-681.9	-670.63
	ARIMAX(0,0,1)×(0,0,1)	Primary,Secondary, Latent, Secondary*Latent	-691.42	-690.33	-676.98
	**ARIMAX(1,0,0)×(0,0,1)**	Primary,Secondary, Latent, Secondary*Latent	**-695.71**	**-694.62**	**-681.27**
	ARIMAX(0,0,1)×(1,0,0)	Primary,Secondary, Latent, Secondary*Latent	-692.54	-691.45	-678.1

Note:

* means the interaction term. The final ARIMAX model selected is highlighted in bold.

### Comparison of ARIMA vs ARIMAX

The contrasts between the observed value of the raw series and the estimated values obtained through the ARIMA model and ARIMAX model were compared. The mean absolute error (MAE), mean absolute percentage error (MAPE), the root mean square error (RMSE) and the mean length of the 95% confidential interval (L95~U95) were selected as measures of evaluation [7]. The result is shown in Table 6. All MAE, MAPE, RMSE and L95~U95 for ARIMAX model are lower than the ARIMA model in the modelling process. The ARIMAX model shows better modelling performance than the ARIMA model. For the point estimation in the testing period, the ARIMAX model for primary, latent and congenital syphilis obtained lower MAE, MAPE and RMSE than the ARIMA model. However the ARIMAX model for secondary and tertiary syphilis had higher MAE, MAPE and RMSE than ARIMA. For the confidential interval estimation, the 95% confidential interval for ARIMAX model are narrower than ARIMA’s, except for latent syphilis.

**Table 6:** Comparison of the performances of the ARIMA model and ARIMAX model.

		MAE		MAPE		RMSE		U95-L95	
		ARIMA	ARIMAX	ARIMA	ARIMAX	ARIMA	ARIMAX	ARIMA	ARIMAX
Modelling Performance	Primary	0.0248	0.0109	0.05	0.022	0.0331	0.0134	0.1317	0.0552
	Secondary	0.0146	0.0114	0.0464	0.0356	0.0199	0.0143	0.0791	0.0644
	Tertiary	0.0013	0.0009	0.1115	0.0771	0.0016	0.0011	0.0063	0.0046
	Latent	0.0469	0.0227	0.0621	0.0294	0.0601	0.0288	0.2389	0.113
	Congenital	0.0032	0.0027	0.0592	0.0492	0.0041	0.0033	0.0163	0.0132
Testing Performance	Primary	0.0754	0.0393	0.1172	0.0598	0.0775	0.0419	0.1476	0.0579
	Secondary	0.0183	0.0201	0.0465	0.0504	0.0195	0.0216	0.0947	0.0782
	Tertiary	0.001	0.0015	0.0534	0.0802	0.0012	0.039	0.0076	0.0046
	Latent	0.1237	0.0795	0.0958	0.0561	0.1463	0.0861	0.2627	0.1815
	Congenital	0.0137	0.0089	0.2054	0.1335	0.0149	0.0097	0.0181	0.0147

## Discussion

Syphilis is a serious public health problem in China. Syphilis is an ulcerative genital disease, which facilitates the transmission of the human immunodeficiency virus (HIV), putting people with syphilis at greater risk for HIV infection [29]. Early recognition of syphilis behavior is of great importance for syphilis control and prevention. The surveillance system is an effective approach to collect and analyze syphilis infection data. Surveillance capacity and reporting practices for syphilis in China have greatly evolved since 2005 [3, 20]. The infection incidence behavior may be precisely detected and estimated with high quality surveillance data. Time series models developed in the current study indicate that disease surveillance data can be utilized to understand the behavior of syphilis over time.

### Modelling of time series

An ARIMA model was used to fit the primary, secondary, tertiary, latent and congenital syphilis univariate time series. The method is widely used in infectious disease areas. The models have been proven powerful in fitting the historical incidence and estimating the incidence in 2012. All syphilis time series obeyed the same pattern, which can be best fitted with the ARIMA(0,0,1)×(0,1,1) model. The pattern is the same as the ARIMA model reported in previous studies [7], also showing that the time series patterns of different types of syphilis are the same under the ARIMA model. An ARIMAX model was fitted to explore the time series correlation among the primary, secondary, tertiary, latent and congenital syphilis time series.

With the inclusion of the covariate variables in the time series, the fitting performance is better than the univariate counterparts. The estimation of confidential intervals of the ARIMAX model are generally narrower than the ARIMA model. The ARIMAX model adds potential prediction value in infection surveillance. The ARIMAX model established in this study effectively captured the trend of the disease. With the ARIMAX model, incidence cannot only be estimated with its historical data, but also the incidence of other relevant diseases, and its influential factors during the surveillance process.

### Seasonality

Decomposition methods were used to extract the seasonality and secular trend of the syphilis time series. Syphilis showed a seasonal and increasing long term trend. The seasonality of secondary syphilis is more obvious than others. The primary, secondary, tertiary and latent syphilis were observed to peak from May to August, while the peak of the congenital disease is later than others, from July to November. May to August is the summer time in China, with warm temperatures and heavier rainfall. Tan et al. found similar seasonality in primary and secondary syphilis peaking from July to September using surveillance data from Chinese Guangdong province [30].

However, there are still some differences in the seasonal patterns among the syphilis’ time series. In particular, secondary syphilis has a greater seasonality oscillation than other time series, which shows a narrower peak and great difference between the peak and the troughs. A possible reason is that secondary syphilis is more severe than other syphilis’ stages, and patients are more likely to seek treatment, making its reporting less likely to be affected by reporting bias.

The seasonality of syphilis epidemic behavior can be related to sexual behaviors in Chinese populations, the impact of seasonal migration in China, and the patients’ clinical attendance [31]. Inferring from the results, the Chinese population would appear to be more sexually active in summer than winter but this might be due to surveillance artefacts. Rural-to-urban migration on an enormous scale in China may expand localized syphilis outbreaks [32]. Labor workers from rural areas usually leave for the city from around March (after the Chinese new year), returning to their rural hometown when winter begins (around late November). Several studies have shown that the rural to urban migrants have higher risk sexual behavior than their rural counterparts [32, 33]. As a result, the government has strengthened the screening of syphilis, especially in high risk populations such as staff in the service industry, drivers, sex workers and drug addicts.

Syphilis serology examination is usually compulsory before marriage, prenatal, for use in the blood supply, to join the army and in regular physical examinations. Graduated students typically look for jobs or enlist in the army in summer.Syphilis’ lower incidence in January and February aligns well with the Chinese Spring Festival (Chinese New Year) when people tend not to visit the clinics for sexual diseases.

### Surveillance artifacts

Many Chinese people with symptoms of a sexually transmitted disease are reported not to seek medical attention [34]. These patients can be found with screening programs. Surveillance artifacts may happen when there is a bias between the trend of surveillance data and the real incidence. When infection happens, patients may not seek medical attention immediately as there are no symptoms. Even when symptoms appear, especially in the primary stage, there are no estimates of how long patients typically wait before seeking advice.

The growth of syphilis incidence in recent years is due to several aspects. The rise of reported syphilis is largely due to the increase of syphilis transmission, which has been analyzed in previous studies. There are some other reasons which may possibly lead to the rise of reported syphilis cases compare to before introducing the new surveillance system. The number of syphilis cases reported in China plateaued for several years before the introduction of the new surveillance system, at which time the number of reported cases began to increase[5]. Sentinel and non-sentinel reporting centers were reported to have different reporting qualities[3]. Therefore, improvements and expansion of the surveillance system around 2004 onwards could partly explain changes in the per-capita number of reported cases. On the other hand, it was reported that very few Chinese people with symptoms of a sexually transmitted disease sought medical attention 10–15 years ago [34].The improvement in clinical attendance of symptomatic patients could also have contributed to part of the rise in reported syphilis cases seen in the current study. The rapid rise of reported syphilis cases can therefore be a combination of increased transmission and surveillance artifacts. However, based on the available data, we cannot estimate the extent of these artifacts.

### Cross-correlations

Generally, the cross correlation among different syphilis incidence time series are very high with no lags, indicating that their changes are synchronous. However, there are no obvious biological or transmission-dynamic explanations for this 0 lag, and this could be due to the aforementioned surveillance artifacts resulting from concentrated testing at particular times of the year. The model established has shown that each type of syphilis can be estimated by including the data of other syphilis series. Primary syphilis progresses to secondary syphilis, and then will evolve into tertiary syphilis without treatment [35]. Secondary syphilis typically occurs 1–2 months after infection, with tertiary syphilis occurs many years after infection. However, the surveillance shows that there are no lags between primary and secondary incidence series. The lag can be covered by the peaking of clinical attendance and passive screening.

### Latent syphilis

Latent syphilis is typically used to describe an asymptomatic person who is found to be positive after months or years of subclinical infection. Latent syphilis accounts for over half of all reported syphilis. Latent syphilis can be detected in various ways, although it is asymptomatic. An investigation on the incidence of latent syphilis among hospitalized patients has shown that 78.83% of latent syphilis patients were detected in the departments of surgery and gynecology, and 12.41% in internal medicine [36]. The incidence of latent syphilis was observed to have increased the fastest among the different types of syphilis, with an increase of 0.01390/100,000 per month after removing seasonality. The incidence of latent syphilis should be monotonically increasing in a real expanding epidemic; this would therefore suggest that any remaining peaks are due to surveillance artifacts.

### Congenital syphilis

Congenital syphilis occurs when the disease reaches the fetus [37]. The incidence of congenital syphilis also reflects seasonal changes. Tan et al. showed that congenital syphilis does not vary seasonally and any seasonal variation is due to reporting biases because pregnant women can acquire syphilis at any point during gestation [30]. However, this may neglect the seasonality of birth rate, which is influenced by social-economic factors [38]. Although there are few reports on the seasonality of birth rate in mainland China, there is research on the seasonality of birth rate among Chinese population in Taiwan, Hong Kong, Malaysia and Singapore, which found that birth rate peaks from September to November [39]. This could explain the increase of congenital syphilis during this period. The lag of congenital syphilis may be dominated by the birth rate trend.

Congenital syphilis has a narrower period with elevated incidence which is likely related to the seasonal pattern of births. In contrast, the seasonality of primary, tertiary, and latent syphilis is much weaker, which is possible due to no significant seasonality (especially for tertiary and latent syphilis) and/or reporting bias.

### Limitation

In addition to the aforementioned limitations found in the data, the limitations of this study are as follows. First, we only obtained syphilis incidence data over an eight-year period and the short length of the syphilis time series may affect the modelling. Second, regional, gender and age data was not considered in the models, which can play an important role in exploring the time series relationship among each type of syphilis. These factors could be included in future studies. Third, the data appear to be affected by a surveillance artifact, which could explain some of the trend in reported syphilis cases. The correlation among each series is the highest with lag 0. This means that we use the same time incidence to estimate other incidences, potentially decreasing the predictive benefit. In ARIMAX, the covariates can become a burden of modelling if the cross-correlations are not high enough. This is a limitation of the method. However, it is useful to establish the relationship between the different syphilis incidence trends, and to forecast the infection behavior. More research is required to exploit the historic incidence of syphilis for the prediction of syphilis’ incidence in other stages.
